# Factors Influencing Colorectal Cancer Screening Participation

**DOI:** 10.1155/2012/483417

**Published:** 2011-12-01

**Authors:** Antonio Z. Gimeno García

**Affiliations:** Department of Gastroenterology, University Hospital of Canary Islands, 38320 Tenerife, La Laguna, Spain

## Abstract

Colorectal cancer (CRC) is a major health problem worldwide. Although population-based CRC screening is strongly recommended in average-risk population, compliance rates are still far from the desirable rates. High levels of screening uptake are necessary for the success of any screening program. Therefore, the investigation of factors influencing participation is crucial prior to design and launches a population-based organized screening campaign. Several studies have identified screening behaviour factors related to potential participants, providers, or health care system. These influencing factors can also be classified in non-modifiable (i.e., demographic factors, education, health insurance, or income) and modifiable factors (i.e., knowledge about CRC and screening, patient and provider attitudes or structural barriers for screening). Modifiable determinants are of great interest as they are plausible targets for interventions. Interventions at different levels (patient, providers or health care system) have been tested across the studies with different results. This paper analyzes factors related to CRC screening behaviour and potential interventions designed to improve screening uptake.

## 1. Introduction

Colorectal cancer (CRC) is the third leading cancer worldwide in terms of incidence accounting for 1.2 million new cases in 2008 (9.7% of total cancers) and the most common malignancy in developed regions (727.000 cases). CRC mortality rates rank fourth after lung, stomach, and liver cancer accounting for 608.000 deaths in 2008 and 8% of all cancer deaths [1].

The efficacy of CRC screening in terms of reduction of incidence and mortality rates has been shown in randomized controlled trials [2–6]. In fact, medical organizations and practice clinical guidelines recommend screening in average-risk population [7–9]. In this way, the most extended CRC screening strategies are based either on annual or biennial faecal occult blood tests (FOBTs), with colonoscopy reserved for patients testing positive, or on endoscopic procedures performed as the primary screening tool performed once only every five years (sigmoidoscopy) or every ten years (colonoscopy). In addition, other screening procedures such as CT colonography and faecal DNA analysis have been recently recommended by some associations [8, 9] although available evidence has been considered insufficient by others [7]. 

Screening uptake, defined as a cross-sectional assessment of compliance is a critical determinant of success for any population-based screening program. High rates of participation has been consistently associated with screening efficacy in terms of mortality reduction as well as cost-effectiveness [10]. This assumption is particularly certain in the case of FOBT-based screening in which recommended intervals are shorter than for other screening strategies (every 1 or 2 years) [11, 12]. 

Recently, a report from the European Commission considered a minimum uptake of 45% in average-risk population as an acceptable goal and 65% as a desirable rate [13], whereas for the American Cancer Society the desirable goal is 75% of the average-risk population [14]. However, despite the available evidence and specific guidelines, CRC screening rates remain far from these aims, although a considerable variability exists around the world. In this way, population-based FOBTs and sigmoidoscopy programmes ranged from 7.2% to 90.1% and from 7% to 55%, respectively, in European countries. In the USA, according to the National Health Interview the proportion of adults older than 50 years who had had a recent screening test ranged from 53% to 73% [14]. However, participation rates for CRC screening are markedly lower than those of other recommended adult preventive services [15]. Therefore, it is important to identify predictors of screening uptake and develop interventional strategies for promoting screening behaviours.

## 2. Predictors of Screening Uptake

Much attention has been given to investigate factors influencing CRC screening participation in average-risk population. A practical way to classify these factors is in non-modifiable factors (i.e., demographics, income, educational level, medical insurance, or family history) and modifiable factors, defined as those susceptible of intervention. Theories of health behaviour or theoretical models have been developed to understand why people do or do not practice different health behaviours, identifying modifiable factors which may be plausible targets of interventional strategies [16, 17]. These factors include knowledge about CRC and screening, perception of risk for developing a CRC, and benefits and barriers against screening or intention to be screened [18]. Therefore, theoretical models have a dual purpose, “explanatory” and “interventionist”. Hereafter, we describe the factors influencing CRC screening.

### 2.1. Sociodemographic Factors

Mixed results have been reported regarding the influence of gender in screening participation. Although, overall, men look to participate more often than women in CRC screening, differences have been found depending on the country and screening strategy. In this way, a recent systematic review [19] showed a higher participation of women in FOBTs-based screening programmes carried out in Europe or Australia. Other studies suggest a higher use of endoscopy among men [20]. In USA, the Behavioral Risk Factor Surveillance System (BRFSS) surveys have consistently reported greater prevalence of CRC test, used among men compared with women [21]. However, generally in pooled analysis data, gender tended to be not significant [10].

Several studies have addressed the association between age and screening uptake [22–26]. In USA [24, 25], screening uptake was superior in elderly (≥65 years) people reporting a peak at 75 years and decreasing around 80–85 years. This finding could be explained, at least in part, because Medicare covers all recommended screening strategies in people older than 65 years, overcoming the economic barrier. However, the same observation was found in a randomized study performed in Italy [27], where men and women aged 65 years or older experienced a significant increase in screening uptake as compared to younger invitees. 

Disparities in screening uptake have been consistently reported in ethnic minorities across the studies [14, 21, 28, 29]. The knowledge of barriers in these groups are of great interest to develop specific intervention strategies. Low income and low educational level have been associated with poor participation rates in minority ethnic groups [28, 29]. These factors could be more important in countries without a universal health coverage. In this regard, screening uptake has been consistently reported lower among minority groups as African Americans or Hispanics in USA [14, 21]. These data contrast with the higher reported incidence and mortality rates observed in African Americans compared with white population (20% and 45% higher incidence and mortality rate of CRC, resp.) [28, 29]. Other factors, such as language difficulties and the expression of culturally influenced health beliefs have been reported in different countries as barriers, independent of the health care system [30, 31]. In this way, information about test procedures and benefits of CRC screening provided by a native-speaking health educator has been suggested as a facilitator for increasing screening uptake in minority ethnic groups [32].

A low socioeconomic status (income, unemployment, educational level, and residence) has been associated with lower screening participation in many studies [14, 21, 33, 34]. This factor is more important in countries in which health services are not government funded. Data coming from the BRFSS surveys [21] consistently report a lower prevalence of CRC screening in those groups with lower hosehold incomes, persons with no health insurance, and unemployed. Lower education, assessed in different studies as less than high school education or having few years of education, has also been reported as a barrier for screening [15, 20, 29] regardless the type of screening strategy used [35]. In an European study carried out in 953 average-risk participants, the ever use of CRC screening being up-to-date screening was more than four times higher among participants with high education level [15]. Although an urban area of residence have been associated with higher rates of screening uptake in US studies [28, 36], contradictory results has been found in Europe [15, 37, 38]. For example, whereas a Swedish study found higher CRC screening uptake in rural areas [37], two Spanish studies [15, 38] did not find any association between willingness to be screened or screening uptake and the area of residence. 

Married people have been shown to be more compliant with healthier behaviour advise elsewhere [39]. In a large European study carried out in UK [40], the authors found that controlling by age and educational level, married couples were more willing to take part in screening programs and presented higher attendance screening rates than those non-married. Invitations of both partners increased screening participation rates. 

Lifestyle and health factors have also been associated with screening uptake. For example, current smoking habit, which has been considered as an indicator of willingness to engage in preventive health behaviour, has been associated with poor CRC screening adherence, whereas screening rates increased in studies reporting participation in former smokers [41, 42]. However, this finding has not been consistently found across the studies [15].

Inconsistent results have also been obtained regarding the effect of comorbidity on screening behavior, and, in consequence, it has been suggested that the effect of specific diseases should be studied separately [41, 43]. Health behaviours such as receiving regular checkups or having a usual source of care have been associated with higher rates of screening uptake [15, 42]. In a nationwide US survey carried out in a representative sample of 61.068 participants aged ≥50 yr [42], routing doctor's visit in the last year was the most important predictive factor of up-to-date CRC screening in the multivariate analysis (OR 3.5, 95% CI (3.2–3.8)) regardless of the screening strategy used. Adherence to other cancer screening behaviours such as prostate cancer screening in men or breast cancer in women has also been positively associated with CRC screening uptake and specific studies have already been carried out [44, 45]. In a large survey study performed in men to investigate the effect of prostate cancer screening in CRC screening uptake [45], adherence to prostate cancer screening exerted the largest independent effect on CRC adherence regardless of the method used for screening (prostate-specific antigen or digital rectal exam) (OR 3.51, CI 95% (3.30–3.73)). Similarly, in the BRFSS, adherence with either cervical cancer or breast cancer screening in women ≥50 yr, was independent predictor of an CRC screening (OR 1.88, *P* < 0.001) [44].

### 2.2. Health Care System and Provider Factors

Health care providers play a key role in the screening behaviour process by increasing awareness about CRC and screening tests in participants, reducing perceived barriers and increasing perceived benefits of screening tests. Physician recommendation has shown a strong correlation with CRC screening behaviours across the studies [46–48]. For example, in a random-digit-dial survey carried out in USA involving 1002 participants ≥50 yr [48], clinician's recommendations were the most important independent predictor of up-to-date CRC screening either in participants were younger or older than 65 yr (OR 13.4, CI 95% (7.2–25) and OR 12.4 CI 95% (5.7–27.1), resp.).

In a recent national representative survey of 1266 US physicians [49], 95% and 80% routinely recommend screening colonoscopy or FOBT to asymptomatic, average-risk patients, respectively. Interestingly, the most frequent practice was to recommend two modalities (56%), with FOBT and colonoscopy being the most commonly-recommended tests (50%). In fact, fewer than 10% routinely recommend all test modalities. This aspect is of great importance as several studies have reported the preferences of average-risk population for different CRC screening tests. Unlike family-risk population for CRC, average-risk population seem to prefer noninvasive testing [50]. Therefore, the clinician's preferences for more invasive tests could be a barrier against screening. Recent evidence suggests that immunochemical FOBT could be better accepted than guaiac occult blood tests because of a lower number of tests required, the lack of dietary and drug restrictions, and easier and less unpleasant sampling methods [51, 52]. Offering available recommended strategies and discussing benefits and drawbacks with patients have been suggested as the most effective procedure to achieve high participation rates [49].

Health system factors have been associated with CRC screening uptake and physician recommendation [53, 54]. Apart from the lack of insurance previously commented, coverage for accessing to the screening service, lack of time to discuss CRC screening with the patient, or lack of physician's reminders have been consistently reported as barriers [53, 54].

### 2.3. Psychosocial Factors

Psychosocial factors involve those related to knowledge about CRC and screening, risk perception of CRC, and perceived barriers and benefits.

#### 2.3.1. Knowledge about CRC and Screening

Knowledge about CRC and screening has been assessed in different ways across the studies [15, 18, 38, 55–57], including questions about risk factors for developing CRC, incidence, prognosis, age-related risk, warning signs or symptoms, and knowledge about recommended CRC screening tests. The lack of knowledge on CRC and screening has been suggested as a prominent barrier to screening adherence [15, 38]. It could be a more important barrier in areas with an opportunistic screening than in those with well-organized programs [58] and it has been reported as a major barrier among minority ethnic groups [59]. In a prospective study carried out in Spain [38], awareness of risk factors (OR 2.32, 95% CI (1.49–3.61); *P* < 0.001) and CRC signs or symptoms (OR 1.65, 95% CI (1.03–2.64); *P* = 0.04) were independent predictors for intention to participate in CRC screening. These authors reported in a later study [15] that knowledge of CRC symptoms was associated with having ever used either CRC procedures (OR 6.46, CI 95% (4.28–9.74); *P* < 0.001) or up-to-date screening (OR 7.23, CI 95% (4.36–11.98); *P* < 0.001).

The relative low public awareness about CRC in European studies contrast with data reported in US population. For example, an Irish study [60] reported that only 26% of patients with CRC could name a CRC symptom, compared with 53% and 71% for lung and breast cancer, respectively. In a recent British population-based sample, recall of cancer warning signs using an open question was less than 30% [61]. In Spain, awareness of at least one warning sign or symptom related to CRC ranged from 21% to 56%. [15, 38]. However, knowledge of CRC screening tests in some states of USA was over 80% [46].

#### 2.3.2. Risk Perception of CRC

High-risk perception of developing CRC have been frequently associated with higher screening participation rates. For example, in one study carried out in a large representative sample of UK, participants who answered that their risk was higher than average-risk population were more willing to participate in CRC screening (98%) than those who answered same risk (84%) or lower risk (74%) [62]. In addition, unhealthy behaviours such as smoking or sedentary has been associated with a higher perception of risk [63, 64]. Similarly, the presence of bowel symptoms, comorbidity, high body mass index, and anxiety has also been associated with increased intention to participate [63, 64]. The lack of recognition of cancer risk has been suggested as a barrier of low participation in cancer screening among nonwhite groups [63]. However, the association between screening uptake and high perception risk has not been consistently found across the studies [15, 38].

#### 2.3.3. Benefits and Perceived Barriers against CRC Screening

Although different theoretical models have been developed in order to achieve a better understanding of health behaviour, all of them identify attitudes as important predictors of intention to screening and screening uptake. One of the most popular theoretical models is the Health Belief Model (HBM) [16]. This model theorizes on people's beliefs regarding the risk for a disease or health problem, and according to their perceptions on the benefits of taking actions to avoid it, analyzes their readiness to take action. In this way, people with negative attitudes such as embarrassment, anxiety, disinterest, fear of cancer or screening, subjective perception of pain or danger about screening, lack of time, feeling healthy, apprehensions about the bowel preparation, laxatives or insertion of a tube, and discomfort are more reluctant to participate in screening programs [18, 56, 65–67]. In a recent study performed in Spain [56], fear to CRC or to screening tests and embarrassment were the main barriers that contributed to a lower participation. This study also suggested that perceived barriers could be more important than benefits in predicting CRC screening. A recent systematic review focused on screening barriers in participants over 65 years found that the most commonly reported barriers related to screening tests were unpleasantness, discomfort, and perceived risk associated with performing tests [68]. Some studies have also suggested that barriers to screening are not homogenous across screening tests and that test-specific barriers warrant consideration in designing strategies to promote screening [69]. 

More barriers have been detected in minority ethnic groups such as African Americans, Asian people, or Hispanics [59, 63, 70]. A recent nationwide study focused on awareness of CRC, and attitudes to sigmoidoscopy screening carried out in UK [59] showed that the most important barrier against screening differed between white and nonwhite participants. Lack of time was the major limiting factor in white participants whereas embarrassment predominated in nonwhite invitees. Attitudes have also been shown to vary depending on socioeconomic position, with negative attitudes overrepresented in lower socioeconomic and less educated groups [18, 71].

## 3. Interventions to Promote CRC Screening Uptake

Interventions aimed at increasing CRC screening uptake can be classified into three categories: those that target patients, those that target providers, and those targeting health systems and communities. 

### 3.1. Interventions Targeting Patients

The benefit of intervention targeting patients, defined as an increment in screening participation, has not always been demonstrated, probably because of the heterogeneity of the studies and several types of interventions used. Patient reminders consist of written or oral information (i.e., phone calls) reminding the necessity of undergoing screening to potential participants [72, 73]. The aim of this intervention is to schedule an appointment with the health care provider in order to demand CRC screening. In general, patient reminder-based studies have shown moderate efficacy for increasing screening uptake [72–75]. In a recent study [72], 1546 participants were randomized to a control group; a standard group (invitation letter, FOBT, and reminder letter); a tailored intervention (standard group intervention and discussion about personal barriers); or a tailored intervention and reminder phone call. One year later, screening uptake was significantly higher in those groups which received reminders compared to the control group (33% versus 46%, 44%, and 48%, resp.). It has also been suggested that the way in which screening is offered to the population may determine screening acceptance. Particularly, two randomized studies have shown that direct mailing of a FOBT kit is an efficient way to increase screening participation in the average-risk population [27, 74]. 

An association has been found between lack of knowledge about CRC and negative attitudes, unwillingness to participate in CRC screening, and finally screening behaviours [18, 56, 76]. Because of the positive relationship between screening uptake and knowledge about CRC and screening, several studies have assessed the impact of educational interventions focused on average-risk screening population. The purposes of these studies are increasing awareness on CRC and screening and motivating people to be screened. It has been suggested that high rates of screening uptake can be achieved by modifying the phases of the “behavior process”, that is, knowledge about the most important features of CRC and screening, attitudes (reducing barriers and increasing perceived benefits), and intention to undergo screening [18, 56]. In some educational interventions participants are provided with some type of educational material including visual images or videotapes [56, 77, 78], educational leaflets [79], posters and calendars [80]. Specific interventions have been designed to increase screening uptake in minority ethnic groups [81, 82]. For example, a patient navigator, defined as a health educator trained in providing better access to healthcare services (i.e., scheduling procedures, educating patients, and explaining instructions for colonoscopy or FOBT) has been demonstrated to be useful for increasing CRC screening uptake in ethnic minority groups [82]. In general a combination of interventions may have a greatest impact on screening rates [83].

### 3.2. Interventions Targeting Providers

Participant's compliance is usually associated with provider's motivation [84]. The aim of the interventions targeting providers is to increase delivery of recommended cancer screening services by providers. Similarly to interventions targeting patients, it has been suggested that reducing barriers and increasing positive attitudes as well as intentions about screening would have a positive impact on screening test recommendation by providers. The desired effect is to stimulate ordering screening tests and finally to increase test completion [85]. Interventions focused on providers include: provider audit and feedback, incentives, and reminders. Regarding provider audit and feedback interventions, medical records are usually analyzed before and after intervention to assess performance of delivering or offering screening tests to patients. A recent systematic review [86] evaluated the effect of this intervention in completion FOBT [87–89] and sigmoidoscopy [88]. Whereas the completion of FOBT screening increased 12 to 23 percentage points, no effect was found in individuals invited to screening sigmoidoscopy. The conclusion was that provider audit and feedback intervention are effective for increasing CRC screening uptake with FOBTs, but the current evidence is insufficient for other screening strategies.

Incentive interventions try to motivate providers with direct or indirect rewards (usually economic incentives) to promote CRC screening in their patients. However, these studies are scarce in the literature and poorly effective [90]. In one study [90], 52 primary care sites were randomized to the intervention or standard care. Intervention consisted of a financial award and an audit and feedback intervention. No significant differences in screening compliance were found between both groups.

Little evidence supports the efficacy of physician reminder-based interventions [91]. In one study [73], 110 physicians and 21.860 patients were randomized to receive reminders or standard care. Whereas screening rates were higher for patients who received mailings compared to those who did not (44.0% versus 38.1%, *P* < 0.001), they were similar among patients of physicians receiving electronic reminders and the standard group (41.9% versus 40.2%, *P* = 0.47).

### 3.3. Organizational Interventions to Improve Access

Improving the referral of patients for screening [92], delivery capacity of services for screening or patient access reducing costs for participants or identifying someone to help patients to navigate the health care system [93] has been associated with an increased screening acceptance. The development of special clinics devoted to screening, the use of planned care screening visits involving physicians and health or non-health professionals could increase screening rates reducing the barrier of physician's lack of time [83]. However, an important financial investment is necessary and it has been reported as a major barrier [94].

## 4. Conclusion

Underuse of population-based CRC screening is a multi-factorial problem involving patients, providers, and the organizational screening process. Plausible target factors for interventions aimed at increasing compliance have been identified at different levels. Specific interventions targeting these factors have been designed to increase screening uptake. However, they have had different success across the studies depending on the screening strategy and the intervention used. Despite the efforts, the impact on screening uptake has been low or moderate. A better knowledge on factors associated with screening compliance and development of more efficient interventions are warranted in order to achieve higher rates of CRC screening uptake.
